# ATLANTIC DIP: The prevalence of pre-diabetes/type 2 diabetes in an Irish population with gestational diabetes mellitus 1-5 years post index pregnancy

**DOI:** 10.1186/1753-6561-6-S4-O35

**Published:** 2012-07-09

**Authors:** C Crowe, E Noctor, LA Carmody, B Wickham, G Avalos, G Gaffney, P O’Shea, F Dunne

**Affiliations:** 1 Department of Medicine, University Hospital Galway and National University of Ireland, Galway, Ireland; 2 Department of Obstetrics and Gynaecology, University Hospital Galway, Ireland; 3 Department of Clinical Biochemistry, University Hospital Galway, Ireland

## Introduction

The ATLANTIC-DIP (diabetes in pregnancy) programme showed 18% of women with gestational diabetes mellitus (GDM) screened with a 75g oral glucose tolerance test (OGTT) 12 weeks post-partum demonstrated glucose intolerance. However, long-term data on progression to type 2 diabetes (T2DM) post gestational diabetes (GDM) in an Irish population is lacking.

## Methods

We compared Caucasian women with previous GDM (n=116), and with normal glucose tolerance (NGT) during pregnancy (n=52), using a 75g OGTT, to determine prevalence of diabetes/pre-diabetes 1-5 years post index pregnancy. Women with abnormal OGTT 12 weeks post-partum (n=22: IFG/IGT, n=20, DM, n=2) did not undergo OGTT, but were included in the analysis. American Diabetes Association diagnostic criteria for IFG/IGT/DM were used.

## Results

Twelve percent (11/94) of GDM patients rescreened had pre-diabetes/DM (IFG/IGT, n=10, DM, n=1), giving a prevalence of 28.4% (33/116) for pre-diabetes/diabetes, versus 2% (1/52) of women with NGT during pregnancy. Logistic regression analysis was used to determine index pregnancy factors associated with post–partum pre-diabetes/diabetes. These were: first-degree relative with DM (OR 2.8 95% CI 1.0,7.4, p=0.04), insulin use during pregnancy (OR 3.4, 95% CI 1.2,9.6, p=0.01), fasting glucose during pregnancy (OR for glucose ≥ 5.6mmol/L: 4.5 95% CI 1.4, 14.2, p= 0.01). and not breastfeeding (OR 3.2 95% CI 1.2, 9.1, p=0.02). BMI in pregnancy was not associated with pre-diabetes/diabetes at 1-5 years.

## Conclusions

The high prevalence of diabetes/pre-diabetes in this population offers an opportunity to develop a screening program to benefit at risk individuals, particularly targeting those with insulin-requiring GDM, higher fasting glucose levels and positive family history.

